# Atractylenolide-I Suppresses Tumorigenesis of Breast Cancer by Inhibiting Toll-Like Receptor 4-Mediated Nuclear Factor-κB Signaling Pathway

**DOI:** 10.3389/fphar.2020.598939

**Published:** 2020-12-08

**Authors:** Fangyi Long, Hong Lin, Xiqian Zhang, Jianhui Zhang, Hongtao Xiao, Ting Wang

**Affiliations:** ^1^ Laboratory Medicine Center, Sichuan Provincial Maternity and Child Health Care Hospital, Affiliated Women’s and Children’s Hospital of Chengdu Medical College, Chengdu Medical College, Chengdu, China; ^2^ Department of Pharmacy, Sichuan Cancer Hospital and Institution, Sichuan Cancer Center, School of Medicine, University of Electronic Science and Technology of China, Chengdu, China; ^3^ Department of Pharmacy, Chengdu Third People's Hospital and College of Medicine, Southwest Jiaotong University, Chengdu, China; ^4^ Department of Breast Cancer, Sichuan Cancer Hospital and Institution, Sichuan Cancer Center, School of Medicine, University of Electronic Science and Technology of China, Chengdu, China

**Keywords:** atractylenolide-I, tumorigenesis, breast cancer, toll-like receptor 4, nuclear factor-κB

## Abstract

**Background:** Toll-like receptor 4 (TLR4) is an essential sensor related to tumorigenesis, and overexpression of TLR4 in human tumors often correlates with poor prognosis. Atractylenolide‐I (AT-I), a novel TLR4-antagonizing agent, is a major bioactive component from *Rhizoma Atractylodes Macrocephalae*. Emerging evidence suggests that AT-I exerts anti-tumor effects on various cancers such as colorectal cancer, bladder cancer and melanoma. Nevertheless, the effects of AT-I on mammary tumorigenesis remain unclear.

**Methods:** In order to ascertain the correlation of TLR4/NF-κB pathway with breast cancer, the expression of TLR4 and NF-κB in normal breast tissues and cancer tissues with different TNM-stages was detected by human tissue microarray and immunohistochemistry technology. The effects of AT-I on tumorigenesis were investigated by cell viability, colony formation, apoptosis, migration and invasion assays in two breast cancer cells (MCF-7 and MDA-MB-231), and N-Nitroso-N-methylurea induced rat breast cancer models were developed to evaluate the anti-tumor effects of AT-I *in vivo*. The possible underlying mechanisms were further explored by western blot and ELISA assays after a series of LPS treatment and TLR4 knockdown experiments.

**Results:** We found that TLR4 and NF-κB were significantly up-regulated in breast cancer tissues, and was correlated with advanced TNM-stages. AT-I could inhibit TLR4 mediated NF-κB signaling pathway and decrease NF-κB-regulated cytokines in breast cancer cells, thus inhibiting cell proliferation, migration and invasion, and inducing apoptosis of breast cancer cells. Furthermore, AT-I could inhibit N-Nitroso-N-methylurea-induced rat mammary tumor progression through TLR4/NF-κB pathway.

**Conclusion:** Our findings demonstrated that TLR4 and NF-κB were over expressed in breast cancer, and AT-I could suppress tumorigenesis of breast cancer via inhibiting TLR4-mediated NF-κB signaling pathway.

## Introduction

Breast cancer, a malignancy stemming from mammary epithelial tissues, stood as the most common cancer in women, and the second contributory factor of cancer related women death all over the world (Siegel et al., 2020). Despite recent improvements in surgical excision, chemotherapy and radiotherapy of breast cancer, patients are often afflicted by various complications such as axillary vein thrombosis, neuropathy and cardiovascular diseases (Meric et al., 2002). Furthermore, the current available treatments with two selective ER modulators (SERMs), tamoxifen and raloxifene, approved by the FDA for breast cancer chemoprevention, remain unsatisfactory due to the drug resistance and side-effects such as hepatic injury (Yang et al., 2013). Therefore, it is crucial to identify the underlying mechanisms involved in tumorigenesis of breast cancer, and facilitate the finding of more effective treatment strategies against breast cancer.

Toll-liked receptors (TLRs), mainly expressing in human immune related cells, plays a crucial role in the first line of host defense by recognizing pathogen-associated molecular patterns (PAMPs) (Kawai and Akira, 2010; O'neill et al., 2013). TLRs could also adjust inflammatory microenvironment, which is vital to tumorigenesis, and multiple effects had been identified by activating TLRs. Various TLRs agonists are currently under investigation for their ability in anticancer immunotherapy (Ray et al., 2014; Sato-Kaneko et al., 2017; Rodell et al., 2019). While several other studies provided evidence that TLRs ligands such as lipopolysaccharide (LPS) was associated with epithelial-to-mesenchymal transition (EMT) and accelerated metastatic tumor growth through TLR4 (Urban-Wojciuk et al., 2019; Rajamanickam et al., 2020). In addition, the expression of TLR4 was the highest among other TLRs in human breast cancer, and TLR4 activation could subsequently activate nuclear factor-κB (NF-κB) and produce pro-inflammatory cytokines, ultimately stimulating inflammation (Yang et al., 2010; Eskiler et al., 2019).

Last few years, naturally existed chemicals have attracted widespread attentions for being identified as new preventive agents against cancer, with high anti-inflammatory efficacy and low toxicity. *Rhizoma Atractylodis Macrocephalae*, one of the traditional Chinese crude materials, had significant gastrointestinal tract protective, neuroprotective and immunomodulatory activities (Ruqiao et al., 2020), and numerous lines of evidence showed that it also exerted anti-tumor, anti-inflammatory and antioxidant effects (Ruqiao et al., 2020; Wu et al., 2020). Atractylenolide‐I (AT-I), the major bioactive component from *Rhizoma Atractylodes Macrocephalae*, also had multiple therapeutic activities including anti-inflammatory (Zhang et al., 2015; More and Choi, 2017) and anti-tumor effects (Fu et al., 2018; Guo et al., 2019; Li et al., 2020). Specifically, AT-I could ameliorate LPS-induced lung damages in mouse model (Zhang et al., 2015), and suppressed LPS-induced NO release and diminished pro-inflammatory cytokines levels in BV-2 cells (More and Choi, 2017). AT-I had the anti-tumor effects against many cancers, such as colorectal cancer (Li et al., 2020), non-small cell lung cancer (Guo et al., 2019) and melanoma (Fu et al., 2018), and it also had a binding site similar to LPS and served as a novel TLR4-antagonizing agent (Liu et al., 2016). Moreover, a randomized pilot study of AT-I on gastric cancer cachexia patients showed that AT-I could inhibit pro-inflammatory cytokines and proteolysis-inducing factor (PIF) proteolysis, and then alleviating symptoms of gastric cancer cachexia patients (Liu et al., 2008). However, the effects of AT-I on mammary tumorigenesis remain largely unknown.

In this study, we investigated the effects of AT-I on mammary tumorigenesis, and explored the possible mechanisms. We found that TLR4 and NF-κB were over expressed in human breast cancer tissues, and activation of TLR4/NF-κB pathway was correlated with advanced TNM-stages. We also found that AT-I could inhibit cell proliferation, migration, invasion and induce apoptosis. Further investigation revealed that AT-I could inhibit TLR4/NF-κB pathway, resulting in decreased proinflammatory factors expression, and these chemopreventive effects of AT-I were TLR4-dependent. Furthermore, *in vivo* studying demonstrated that AT-I suppressed tumorigenesis of breast cancer via inhibiting TLR4/NF-κB pathway. All these results suggested that AT-I could be a potential agent in suppressing tumorigenesis of breast cancer.

## Material and Methods

### Cell Lines and Reagents

The human breast cancer cell lines (MCF-7 and MDA-MB-231) and mammary epithelial cell line (MCF 10A) were obtained from the American Type Culture Collection (Manassas, VA, USA). Atractylenolide-I (AT-I, purity ≥98%) was purchased from Solarbio (Beijing, China). MTT [3-(4, 5-dimethylthiazol-2-yl)-2, 5-diphenyl tetrazolium bromide] was from Sigma-Aldrich (St. Louis, MO, USA). NF-κB inhibitor 4-N-[2-(4-phenoxyphenyl)ethyl]quinazoline-4,6-diamine (QNZ) was from Selleckchem (Houston, TX, USA). The primary antibodies used in this study, targeting TLR4, MyD88, p-NF-κB p65, NF-κB p65, p-IκBα, IκBα, p-IKKα/β, IKKα, IKKβ, procaspase-3, cleaved caspase-3, PARP, cleaved PARP, E-cadherin, vimentin and β-actin are listed in Table 1. Recombinant retroviral GFP vector harboring a short-hairpin RNA (shRNA) sequence targeting TLR4 (shTLR4) and its scramble vector (shNC) were purchased from OriGene (Rockville, MD, USA). Annexin V-APC/PI apoptosis detection kit was from Keygen (Nanjing, China). The human and rat ELISA kits for interleukin-6 (IL-6), interleukin-1β (IL-1β) and tumor necrosis factor-alpha (TNF-α) were purchased from Dakewe (Shenzheng, China).

**TABLE 1:** Primary antibodies used in this study.

Antibody	Description	Application	Source
TLR4	Transmembrane receptor	WB, IHC	Santa cruz
MyD88	Myeloid differentiation protein	WB	Santa cruz
p-NF-κB p65	Activated transcription factor	WB	Santa cruz
NF-κB p65	Transcription factor	WB, IHC	Santa cruz
p-IκBα	Activated inhibitory protein of NF-κB	WB	Cell signaling technology
IκBα	Inhibitory protein of NF-κB	WB	Cell signaling technology
p-IKKα/β	Activated IκB kinase	WB	Cell signaling technology
IKKα	IκB kinase	WB	Cell signaling technology
IKKβ	IκB kinase	WB	Cell signaling technology
Procaspase-3	Precursor form of caspase-3	WB	Abcam
cleaved caspase-3	Active form of caspase-3	WB	Abcam
PARP	Cleavage targets of caspase-3	WB	Abcam
Cleaved PARP	Cleavage of PARP	WB	Abcam
E-cadherin	Intercellular junction protein	WB	Abcam
Vimentin	Cytoskeletal protein	WB	Abcam
β-actin	Cytoskeletal protein	WB	Boster

WB, Western blot; IHC, Immunohistochemistry.

### Tissue Microarrays and Immunohistochemistry

Commercial tissue microarray (Alenabio Biotechnology, Xi'an, China), composed of 8 samples of normal or cancer-adjacent breast tissues and 40 samples of breast cancer tissues, was used to evaluate the expression of TLR4 and NF-κB p65. The tumor tissues included 9 cases of Stage I disease, 25 cases of Stage II disease and 6 cases of Stage III disease.

Immunohistochemistry was performed on the human breast tissue microarray. The slides were deparaffinized and rehydrated in graded ethanol solutions. After wet autoclave pretreatment for antigen retrieval and suppressing endogenous peroxidase activity, the slides were blocked with 5% bovine serum albumin (BSA). The anti-TLR4 monoclonal antibody (1:100) and NF-κB p65 (1:100) were applied for 1 h at 37 °C, and SABC Staining System kit (Boster, Wuhan, China) containing secondary antibody was used. Immunoreactivity was visualized using 3,3'-diaminobenzidine (Boster, Wuhan, China). Immunohistochemistry evaluation was performed independently by two researchers. A semi-quantitative HistoScore (H-score, ranged from 0 to 300) for each specimen was calculated by multiplying the percentage of positive areas (0–100%) by intensity (0 = nil; 1 = weak; 2 = moderate; 3 = strong) (Rojo et al., 2016).

### Cell Culture and Transfection

MCF-7 and MDA-MB-231 cells were cultured in Dulbecco’s Modified Eagle’s Medium (Gibco, Carsbad, CA, USA) supplemented with 10% fetal bovine serum (Gibco) and 100 U/ml of penicillin and 100 μg/ml streptomycin, and at 37 °C in a 5% CO_2_ atmosphere. MCF 10A cells were cultured in DMEM/F12 (Gibco) supplemented with 5% heat-inactivated horse serum (Gibco), 100 ng/ml Cholera toxin, 10 μg/ml insulin, 20 ng/ml recombinant EGF, 0.5 μg/ml hydrocortisone, 100 U/ml of penicillin and 100 μg/ml streptomycin at 37 °C in a 5% CO_2_ atmosphere.

### Cell Viability Assay

MCF 10A, MCF-7 and MDA-MB-231 cells viability were measured by MTT assay. The cells were seeded into 96-well plates (1×10^4^ cells/well) and incubated for 24 h. After treatment with AT-I (0, 25, 50, 100 and 200 μM) for 24, 48 and 72 h or QNZ (50 nM) for 48 h, the cells in each well were incubated with 10 μl MTT (5 mg/ml) for 4 h at 37 °C, and then MTT was dissolve with 150 μl DMSO. The absorbance was measured using a Multiskan MK3 Reader (Thermo Fisher Scientific, Waltham, MA, USA) at 570 nm wavelength.

### Protein Lysate Preparation and Western Blot Assay

The total protein was isolated from cell lysates using RIPA buffer (Beyotime, Shanghai, China) according to the manufacturer's instructions. Equal amounts of protein were separated by gel electrophoresis, and then transferred to a polyvinylidene fluoride membrane (Bio-Rad, Hercules, CA, USA). After blocked with 5% BSA, the membrane was incubated with primary antibody (Table 1) overnight at 4 °C, washed three times, and subsequently incubated with horseradish peroxidase-conjugated secondary antibody (ZSGB, Beijing, China) for 2 h at 25 °C. The density analysis of each band was conducted using Image Lab 5.0 software (Bio-Rad).

### Annexin V-Allophycocyanin/Propidium Iodide Staining and Apoptosis Assay

Annexin V-APC and propidium iodide (PI) staining was used to detect the effects of AT-I on apoptosis of MCF-7 and MDA-MB-231 cells. Briefly, the cells were seeded in 6-well plates (3×10^5^ cells/well) and treated with AT-I (0–100 μM) or vehicle (0.1% DMSO) for 48 h, respectively. The cells were then trypsinized, washed twice with ice-cold PBS, re-suspended and incubated with binding buffer that containing Annexin V-APC and PI labelling reagents at room temperature for 15 min in the dark. Early apoptotic cells [Annexin V-APC-positive and PI-negative (Ann+/PI−)] and late-stage apoptotic [dual Annexin V-APC-positive and PI-positive (Ann+/PI+)] were examined by flow cytometer. Simultaneously, we detected the protein expression of procaspase-3, cleaved caspase-3, PARP, cleaved PARP in MCF-7 and MDA-MB-231 cells after AT-I treatment by western blot assay.

### Cell Migration Assay

Wound healing assay was adopted to detect the migration abilities of MCF-7 and MDA-MB-231 cells. Briefly, the cells were seeded and grew to 80% confluence in 24-well plates, and we created a wound by scratching cells with a sterile 200 µL pipette tip. Then, cells were continued to incubate in the medium in the absence or presence of 25, 50 and 100 µM AT-I for 48 h. The cells grew into wound surface were considered as migrated cells, and photographed by a microscope. The wound width difference of 0 and 48 h was used to calculated the rate of wound healing.

### Cell Invasion Assay

Transwell chambers (Millipore, Billerica, MA, USA) with 8.0 μm pore membranes were coated with matrigel (BD, Franklin Lakes, NJ, USA) and used for invasion analysis. In brief, cells were treated with 0–100 µM AT-I. After for 48 h, cells were collected by trypsin and resuspended in 200 μL serum-free medium, and then seeded on the upper chamber. 600 μL complete medium was added to the lower chamber as a chemoattractant. After 24 h incubation, the cells remaining at the upper surface of the membrane were removed, and the cells on the lower surface, regarded as invasive cells. After fixing with 4% paraformaldehyde, the cells were stained with 0.5% crystal violet solution. The invasive cells were photographed and counted under microscope.

### Colony Formation Assay

MCF-7 and MDA-MB-231 cells were seeded in 6-well plates, and cultivated in medium containing AT-I (0–100 µM) for 48 h. Cells were maintained in the well for 14 days to form colony, and the colonies were fixed and stained with 0.5% crystal violet solution (30% methanol) for 30 min at room temperature. The numbers of colonies with ≥50 cells were counted under microscope.

### Animal and Experiments

Twenty-four female Sprague-Dawley (SD) rats (Dashuo, Chengdu, China) were randomly distributed into four groups. After pretreatment with vehicle or AT-I (100 and 200 mg/kg) for 3 days, a single dose of NMU (75 mg/kg) was intraperitoneal injected to the rats at 21 days of age, and continued to treat with vehicle or AT-I daily for 9 weeks. Then they were sacrificed, and cancer samples were collected and kept at −80 °C for western blot and ELISA analysis.

### Enzyme Linked Immunosorbent Assay

For measurements of TNF-α, IL-6 and IL-1β, the supernatants of cell culture medium and rat tissue lysis were collected to performe ELISA analysis according to the manufacturer’s protocol.

### Statistical Analysis

The data was expressed as the mean ± SEM and analyzed using one-way analysis of variance (ANOVA) followed by the Tukey test. The statistical analysis was performed using SPSS 16.0 software. Values of *p* < 0.05 were considered statistically significant.

## Results

TLR4 and NF-κB were up-regulated in human breast cancer tissues and correlated with advanced TNM-stages.

**FIGURE 1:**
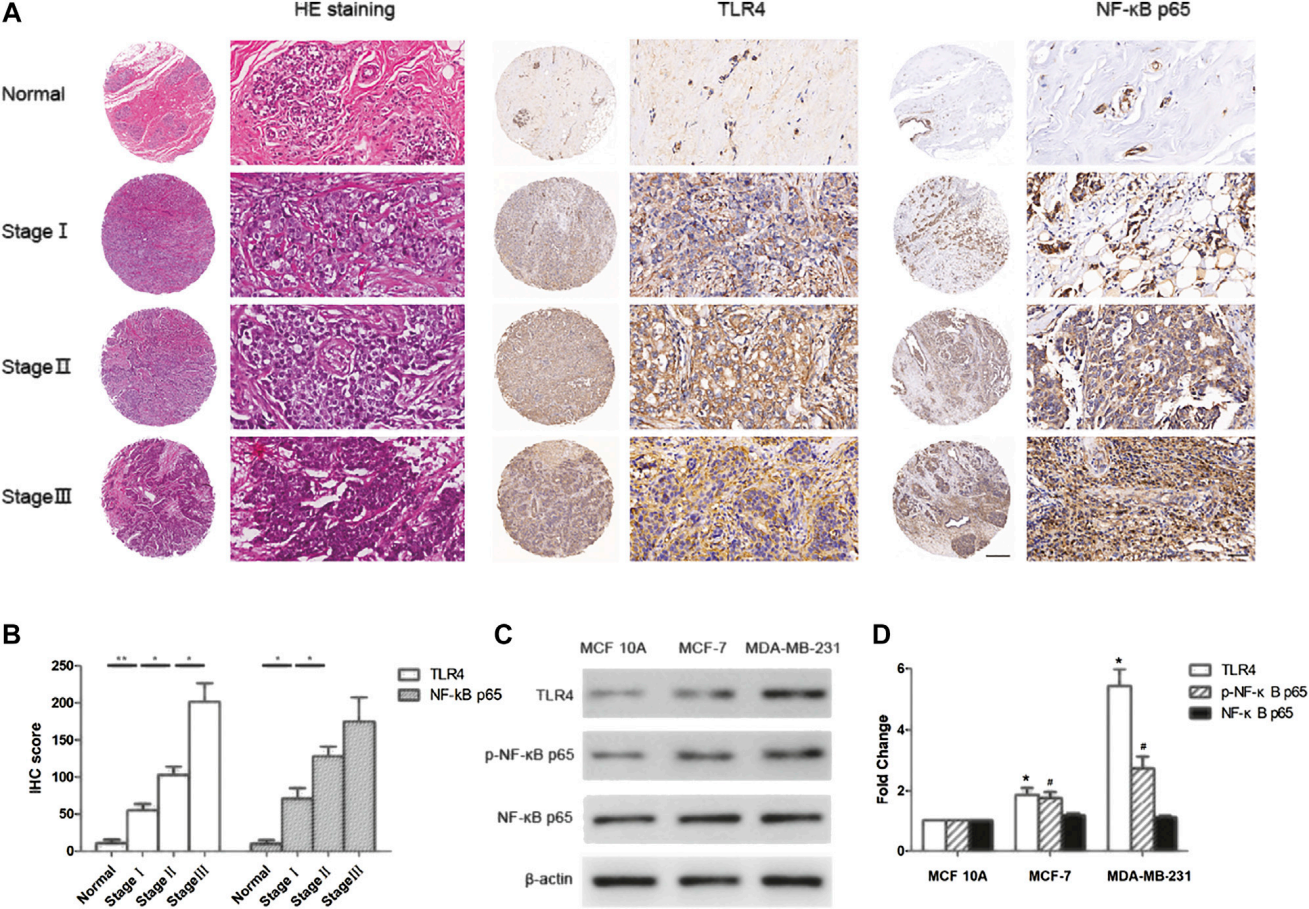
TLR4 and NF-κB were over expressed in breast cancer and correlated with TNM stages. **(A, B)** Representative immunohistochemistry staining for TLR4 and NF-κB p65 in normal breast tissues and breast cancer tissues with different TNM stages. *p < 0.05, **p < 0.01 between different groups. **(C, D)** The expression of TLR4, p-NF-κB p65 and NF-κB p65 in MCF 10A, MCF-7 and MDA-MB-231 cells by western blot. Significant differences between different groups were indicated as *p < 0.01, vs. MCF 10A group, n = 3.

To study the role of TLR4/NF-κB pathway in breast cancer, we assessed the expression of TLR4 and NF-κB in normal breast tissues and different TNM-stages breast cancer tissues using tissue microarray and immunohistochemistry technology. The results showed that TLR4 and NF-κB levels in breast cancer tissues were significantly higher than that in normal breast tissues (Figures 1A,B). Furthermore, we found that the expression of TLR4 and NF-κB in high TNM stages was significantly higher than that in low TNM-stages of breast cancer (Figures 1A,B). In addition, we detected the expression of TLR4 and NF-κB in MCF-7, MDA-MB-231 and MCF 10A cells by western blot assay. As shown in Figures 1C,D, TLR4 and NF-κB levels in breast cancer cells were significantly higher than that in mammary epithelial cells. These results indicated that the expression of TLR4 and NF-κB were up-regulated in human breast cancer tissues and cells, and their high expression was correlated with advanced TNM-stages in breast cancer patients.

### Atractylenolide‐I Inhibited Breast Cancer Cell Growth and Induced Apoptosis

**FIGURE 2:**
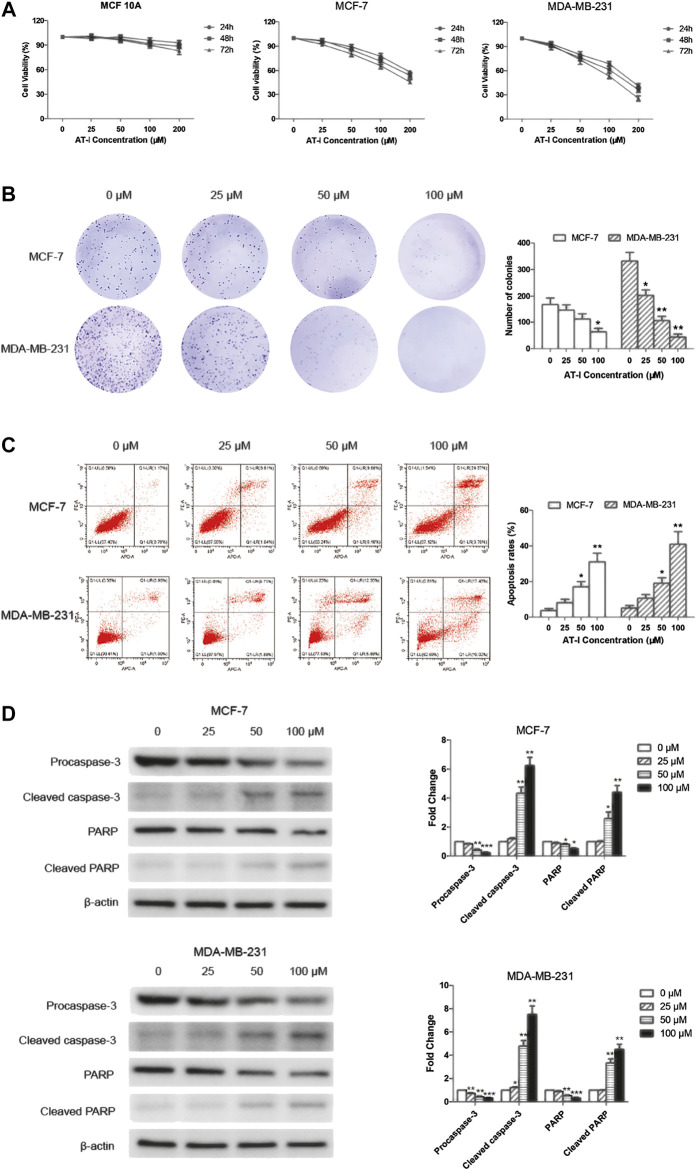
AT-I inhibited cell growth and induced apoptosis in breast cancer cells. **(A)** MCF 10A, MCF-7 and MDA-MB-231 cells were treated with AT-I (0–200 µM) for 24, 48 and 72 h to determine cell viability. **(B)** MCF-7 and MDA-MB-231 cells were treated with AT-I (0–100 µM) for 48 h and then applied for colony formation. The number of colonies was counted after 14 days. **(C)** MCF-7 and MDA-MB-231 cells apoptosis assay after treatment with AT-I (0–100 µM) for 48 h. **(D)** The effects of AT-I on the expression of procaspase-3, cleaved caspase-3, PARP and cleaved PARP were measured by western blot assay. Significant differences between different groups were indicated as *p < 0.05, **p < 0.01, ***p < 0.001, vs. the 0 μM control group, n = 3.

Cell viability was determined by MTT assay after AT-I treatment, and the results (Figure 2A) demonstrated that AT-I cytotoxicity was dose- and time-dependent in both MCF-7 and MDA-MB-231 cells, with the IC50 values (251.25 ± 27.40) μM, (212.44 ± 18.76) μM and (172.49 ± 18.32) μM for 24, 48 and 72 h in MCF-7 cells, respectively; and (164.13 ± 17.90) μM, (139.21 ± 17.67) μM and (105.68 ± 10.58 μM) in MDA-MB-231 cells, respectively. However, no obvious cytotoxicity was detected in MCF 10A cells after incubated with different concentrations of AT-I (0–200 µM) for 24–72 h. Furthermore, there was a significant inhibition of colony formation after AT-I treatment (Figure 2B), suggesting that AT-I could inhibit cell proliferation and tumorigenic ability of MCF-7 and MDA-MB-231 cells.

To further investigate the anti-tumor activities of AT-I, we tested effects of AT-I on cell apoptosis. The MCF-7 and MDA-MB-231 cells were treated with AT-I at different concentrations for 48 h. The percentage of live cells (Q1-LL), early apoptotic (Q1-LR), late apoptotic (Q1-UR), and necrotic (Q1-UL) of MCF-7 and MDA-MB-231 cells were determined by flow cytometry. The results indicated that AT-I could induce apoptosis in a dose-dependent manner in both MCF-7 and MDA-MB-231 cells (Figure 2C). To further investigate the mechanism of apoptosis induced by AT-I, we detected the cleaved fragments of caspase-3 and PARP, the well-known molecular markers of apoptosis (Elmore, 2007) in MCF-7 and MDA-MB-231 cells after 48 h AT-I treatment by western blot assay. The results showed that AT-I triggered caspase-3 and PARP cleavage as seen by the reduced expression of procaspase-3 and full length PARP and increased expression of cleavage of caspase-3 and PARP (Figure 2D).

### Atractylenolide‐I Inhibited Migration and Invasion of Breast Cancer Cells

**FIGURE 3:**
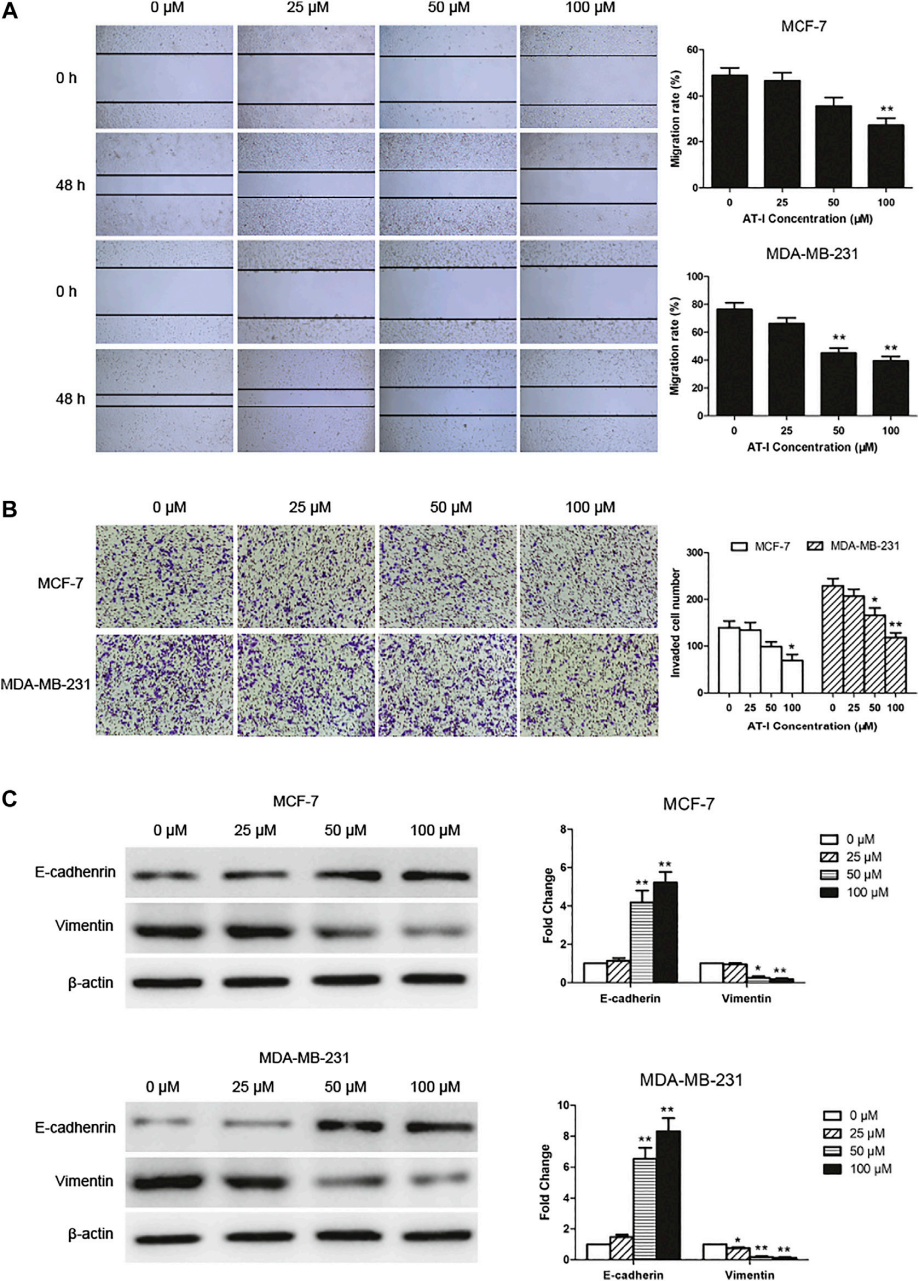
AT-I inhibited cell migration and invasion. **(A)** The migration capability of MCF-7 and MDA-MB-231 cells were calculated by wound healing assay after treatment with AT-I (0–100 µM) for 48 h (40×magnification). **(B)** Transwell invasion assay showed the number of invaded cells in MCF7 and MDA-MB-231 cells treated with different concentrations of AT-I (0–100 µM) for 48 h (100×magnification). **(C)** The effects of AT-I on the expression of E-cadherin and vimentin were measured by western blot assay. Significant differences between different groups were indicated as *p < 0.05, **p < 0.01, vs. the 0 μM control group, n = 3.

We explored the effects of AT-I on cell migration and invasion, and the wound healing assay suggested that the migration of MCF-7 and MDA-MB-231 cells was significantly inhibited by 50 or 100 μM AT-I treatment for 48 h (Figure 3A). The transwell invasion assay showed that the number of cells invading the lower chamber was markedly decreased after 50 or 100 μM AT-I treatment, compared with the control cells (Figure 3B). In addition, as dose increased, AT-I has stronger inhibitory effects on the migration and invasion of MCF-7 and MDA-MB-231 cells. Furthermore, western blot assay showed that AT-I could up-regulate the expression of E-cadherin and down-regulate the expression of vimentin. Taken together, these results strongly demonstrated that AT-I treatment resulted in effective inhibition of migration and invasion in breast cancer cells.

### Atractylenolide‐I Inhibited Toll-like receptor 4/Nuclear Factor-κB Pathway in Breast Cancer Cells

**FIGURE 4:**
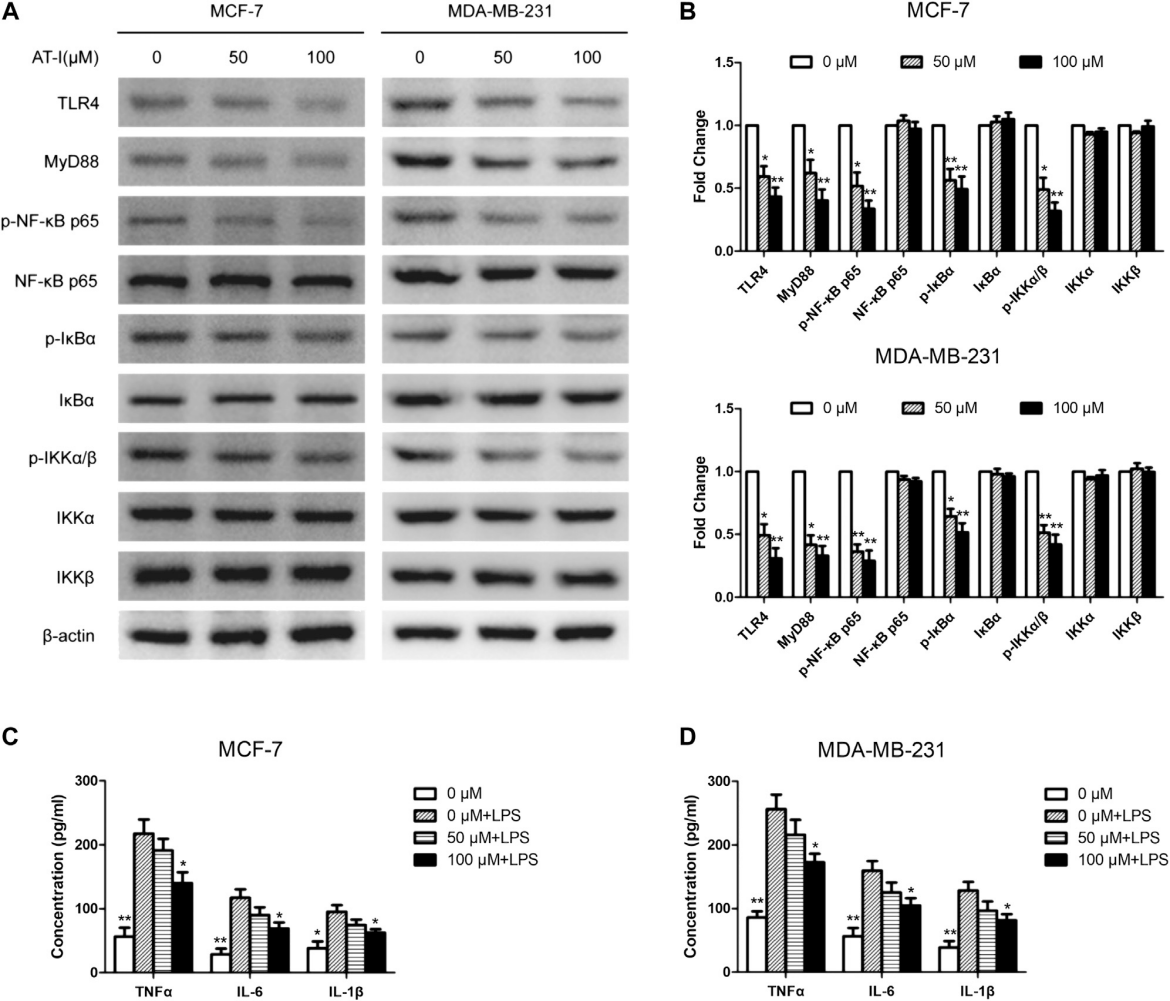
AT-I regulated TLR4/NF-κB signaling pathway in breast cancer cells. **(A, B)** The effects of AT-I on TLR4/NF-κB signaling pathway were measured by western blot assay. **(C, D)** The levels of TNF-α, IL-6 and IL-1β in the supernatants of cells were measured by ELISA assay. Significant differences between different groups were indicated as *p < 0.05, **p < 0.01, vs. the 0 μM control group or the LPS treated control group, n = 3.

AT-I was reported to be a novel TLR4-antagonizing agent (Liu et al., 2016), and we used western blot to detect the effects of AT-I on TLR4/NF-κB pathway in MCF-7 and MDA-MB-231 cells. After AT-I treatment, the expression of TLR4, MyD88, p-NF-κB p65, p-IκBα and p-IKKα/β was significantly down-regulated in a dose-dependent manner in both cells (Figures 4A,B). Furthermore, we used LPS (TLR4 agonist) induced cell inflammation to measure the levels of NF-κB related pro-inflammatory cytokines by ELISA assays. The results showed that the levels of the NF-κB-regulated cytokines (i.e., TNF-α, IL-6 and IL-1β) were all decreased after AT-I treatment. These data suggested that AT-I could inhibit TLR4/NF-κB pathway, and down-regulate the downstream pro-inflammatory cytokines in breast cancer cells (Figures 4C,D).

The tumorigenesis inhibitory effects of AT-I was mediated by TLR4.

**FIGURE 5:**
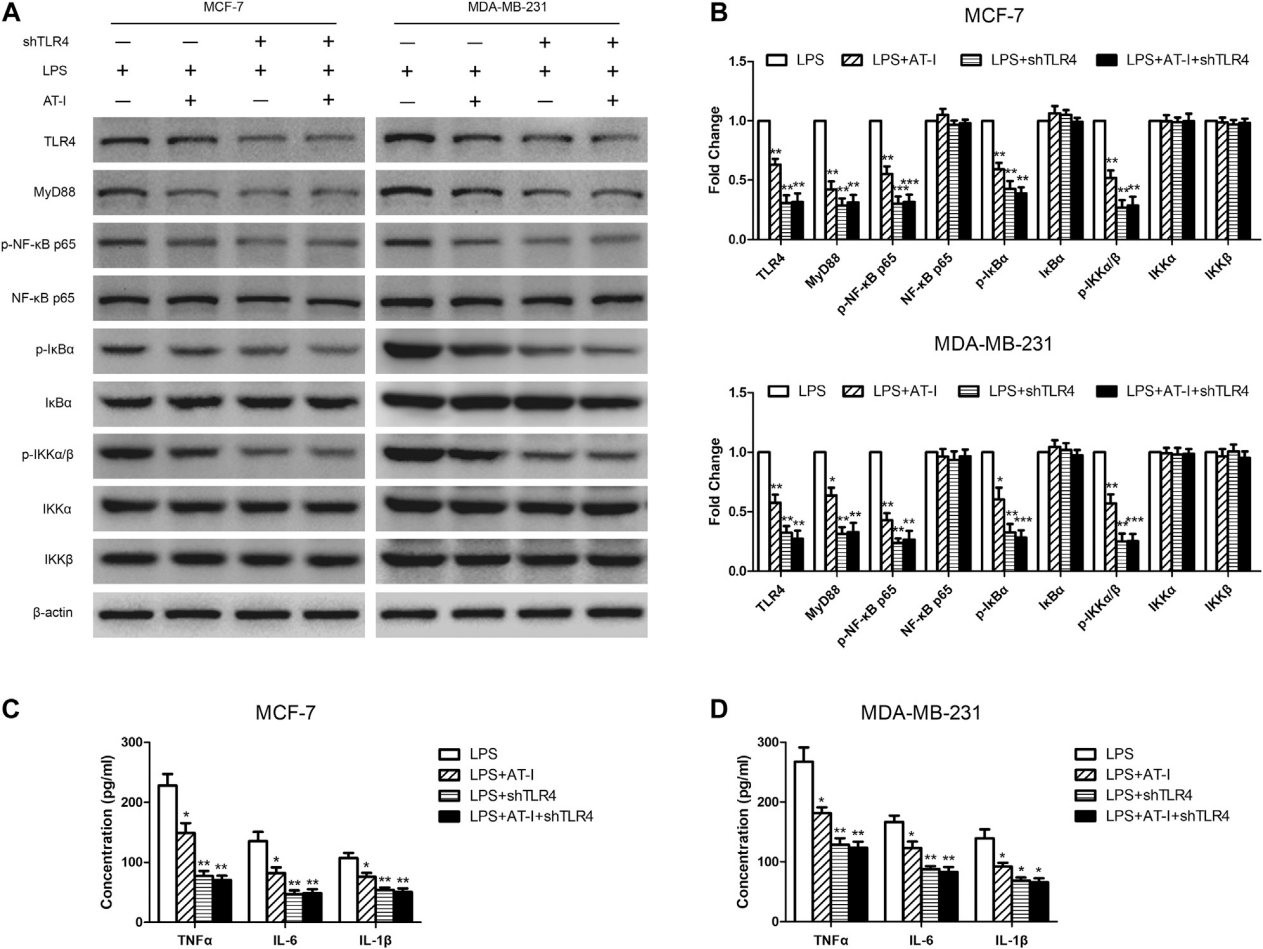
The effects of AT-I on suppressing tumorigenesis was mediated by TLR4. The shTLR4 plasmid was used to transfect breast cancer cells, and then western blot and ELISA assays were used to compare related proteins expression with untransfected cells after cells had been pre-treated in presence or absence AT-I for 48 h during LPS stimulation. **(A, B)** The expression of TLR4/NF-κB signaling pathway was detected by western blot assay. **(C, D)** The levels of TNF-α, IL-6 and IL-1β in the cell supernatants were measured by ELISA assay. Significant differences between different groups were indicated as *p < 0.05, **p < 0.01, ***p < 0.001, vs. the LPS treated control group, n = 3.

To confirm that TLR4 was essential in the tumorigenesis inhibitory effects of AT-I, the shTLR4 plasmid was adopted to transfect MCF-7 and MDA-MB-231 cells, and then western blot and ELISA assays were used to evaluate the changes of related protein levels after AT-I treatment. We found that the expression of TLR4, MyD88, p-NF-κB p65, p-IκBα and p-IKKα/β were significantly down-regulated after AT-I or shTLR4 treatment in LPS-induced MCF-7 and MDA-MB-231 cells. However, no inhibitory effects of AT-I on these protein levels were observed after shTLR4 transfection in LPS induced cells (Figures 5A,B). To clearly demonstrate the mechanisms, the levels of secreted pro-inflammatory cytokines TNF-α, IL-6 and IL-1β were determined by ELISA, and the results were consistent with western blot assays (Figures 5C,D).

**FIGURE 6:**
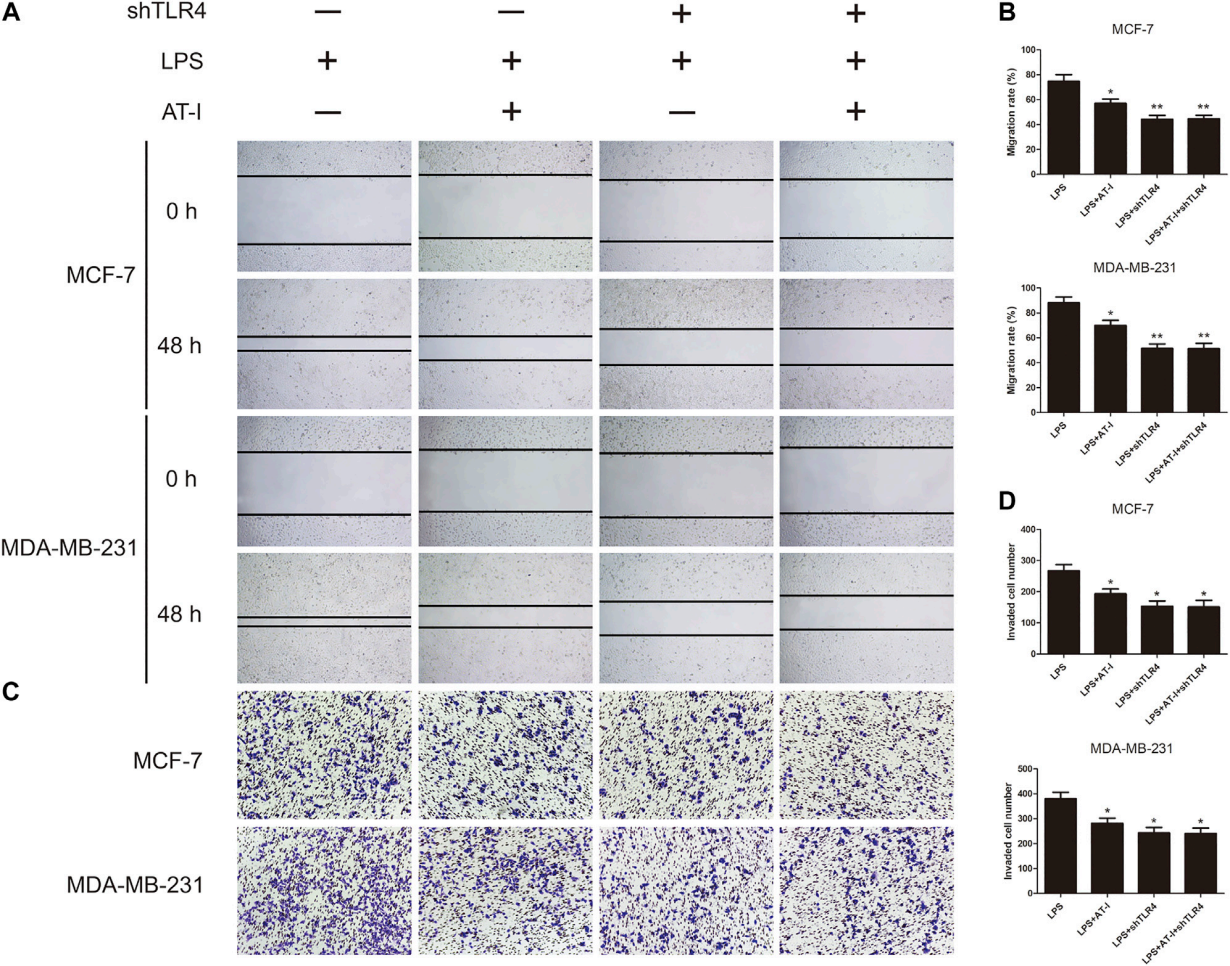
The effects of AT-I on cell migration and invasion was mediated by TLR4. LPS induced shTLR4 transfected and control cells were treated with AT-I, and then seeded in 24-well plates or transwell chamber coated with matrigel. **(A, B)** The migration capability were calculated by wound healing assay (40×magnification). **(C, D)** The invasion capability were calculated by transwell invasion assay (100×magnification). Significant differences between different groups were indicated as *p < 0.05, **p < 0.01, vs. the LPS treated control group, n = 3.

To further examine the role of TLR4 in the migration and invasion of breast cancer cells after AT-I treatment, the LPS induced MCF-7 and MDA-MB-231 cells were used for wound healing and transwell invasion assays. The results indicated that LPS could induce the migration and invasion activities of both MCF-7 and MDA-MB-231 cells, and treatment with AT-I or shTLR4 could decrease cell migration and invasion. However, in TLR4 knockdown cells, no inhibitory effects of AT-I on cell migration and invasion were observed, which indicated that TLR4 is essential for AT-I’s inhibitory effects on cell migration and invasion (Figures 6A–D).

### Atractylenolide‐I Inhibited N-Nitroso-N-methylurea-Induced Mammary Tumor Progression in Rats

**FIGURE 7:**
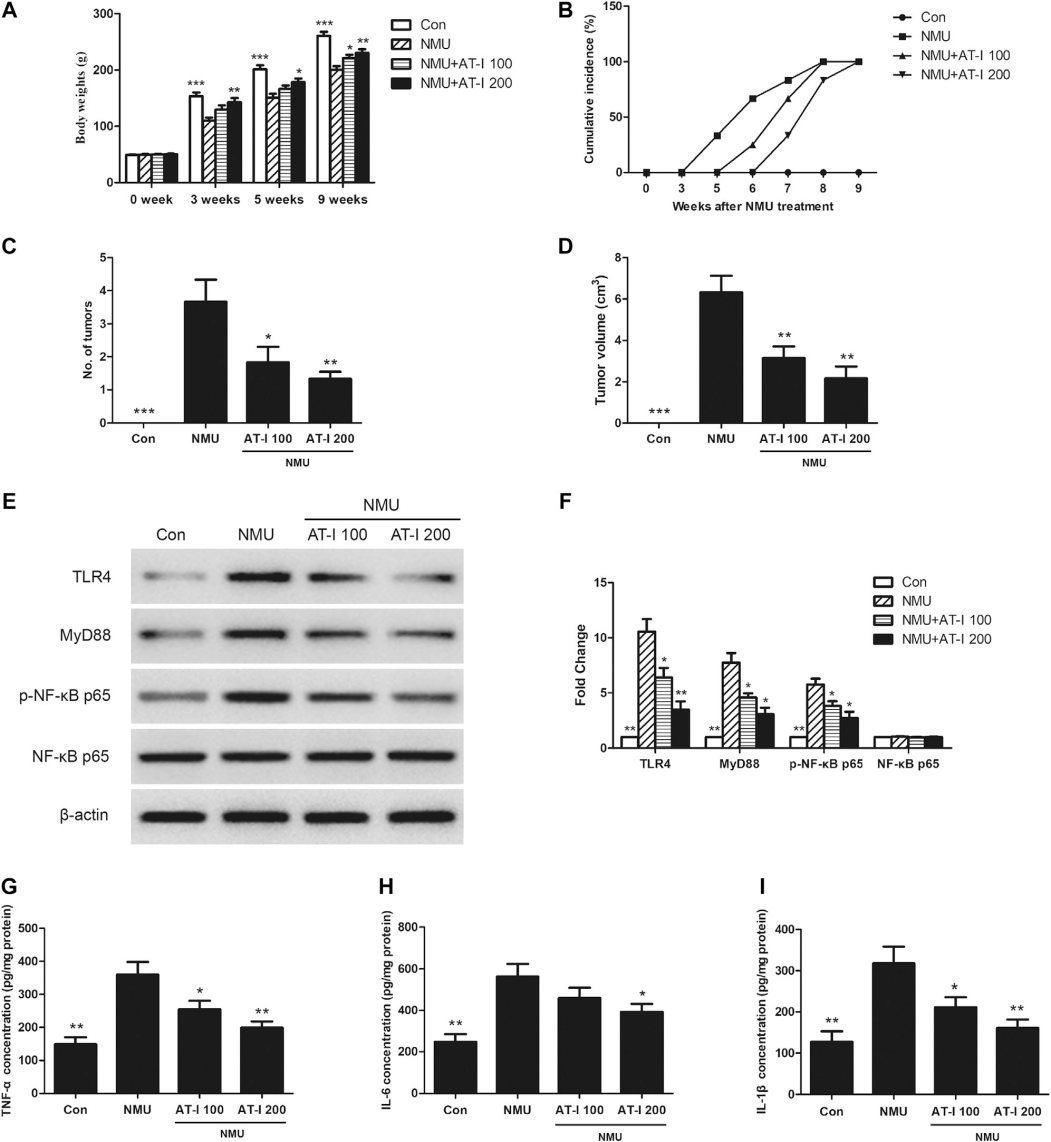
AT-I inhibited NMU-induced rat breast cancer. The NMU-induced rats were treated with control or AT-I for 9 weeks. **(A)** Body weights of rats in all groups at 0, 3, 5 and 9 weeks. **(B)** Palpable tumor incidence was recorded every week. **(C)** Average tumor number at 9 week. **(D)** Average tumor volume at 9 week. **(E, F)** Rat breast tissues were kept to detect TLR4/NF-κB pathway by western blot assay at the end of experiment. **(G, H, I)** TNF-α, IL-6 and IL-1β levels in rat breast tissues were analyzed by ELISA at the end of experiment. Significant differences between different groups were indicated as *p < 0.05, **p < 0.01, ***p < 0.001, vs. the NMU group, n = 6.

The previous studies indicated that AT-I exerted tumorigenesis inhibition *in vitro*, and we next investigated these effects *in vivo*. We employed the NMU-induced rat breast cancer model, which is commonly accepted for candidate chemopreventive agents evaluation [23, 24]. We firstly found that NMU treatment could significantly decrease the body weights of rats, while AT-I could revert these effects (Figure 7A). In addition, the first palpable mammary tumors in the NMU-treated group appeared after 5 weeks of NMU treatment, but it did not appear until 6 and 7 weeks in 100 mg/kg and 200 mg/kg AT-I group, and all rats had tumors at 9 weeks (Figure 7B). Averagely there were 3.67, 1.83 and 1.33 tumors monitored in the NMU, 100 and 200 mg/kg AT-I treatment groups, respectively (Figure 7C). Moreover, the mean tumor volume was significantly decreased in the 100 and 200 mg/kg AT-I treatment group (Figure 7D). These results indicated that AT-I treatment could inhibit the mammary tumorigenesis in rats.

To further elucidate the underlying mechanisms of these effects, we detected the influence of AT-I on the TLR4/NF-κB pathway and its downstream proinflammatory factors in the NMU-induced mammary carcinogenesis. As shown in Figures 7–I, NMU treatment alone could induce TLR4, MyD88 and p-NF-κB p65 expression, and then increase its downstream proinflammatory factors TNF-α, IL-6 and IL-1β levels. However, AT-I treatment could reduce the activation of TLR4/NF-κB pathway induced by NMU, and then decrease the TNF-α, IL-6 and IL-1β level. Our findings suggested that AT-I could inhibit NMU-induced mammary tumor progression in rats through inhibiting TLR4/NF-κB pathway.

## Discussion

It is well established that tumorigenesis is a multi-step process caused by various factors, such as environmental carcinogens, inflammatory mediators, and tumor promoters. Multiple effects had been identified of TLR4 in tumor progression (Yang et al., 2010; Liao et al., 2012; Li et al., 2017; Khademalhosseini and Arababadi, 2019; Zandi et al., 2019). In breast cancer cells and primary breast cancer tissues from patients, TLR4 expression was found to be up-regulated both at mRNA and protein levels, and significantly correlated with the high incidence of lymph node metastasis (Yang et al., 2010; Zandi et al., 2019). Moreover, as TLRs ligands, LPS was reported to increase breast cancer metastasis *in vitro* and *in vivo* (Li et al., 2017), and acquiring of high metastatic potential upon the TLR4-elicited activation of NF-κB in breast cancer cells was associated with integrin αvβ3, TPM1 and maspin (Liao et al., 2012). However, Connelly et al. suggested that inhibition of NF-κB might lead to the increased tumor latency and decreased tumor burden and numbers of lung metastases during breast cancer development in mice (Connelly et al., 2011). All these studies suggested that TLR4/NF-κB pathway played a critical role in the mammary tumorigenesis, but the underlying mechanisms need to be further elucidated.

To study the important role of TLR4/NF-κB pathway in breast cancer, we assessed the expression of TLR4 and NF-κB among normal breast tissues and different TNM-stages breast cancer tissues using tissue microarray and immunohistochemistry technology. The results showed that TLR4 and NF-κB were over expressed in breast cancer, and correlated with the TNM-stages. Furthermore, these differentially expressed levels of TLR4 and NF-κB were also detected in breast cancer cells and mammary epithelial cells. These results indicated that TLR4 and NF-κB were up-regulated in breast cancer, and might function as tumor promoters in breast cancer.

**FIGURE 8:**
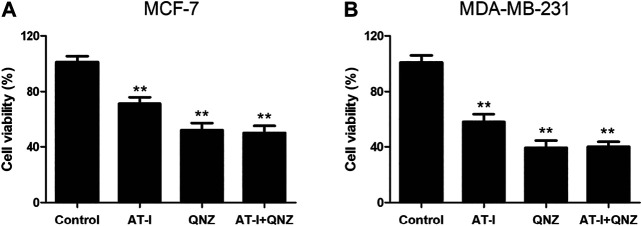
Effects of AT-I and QNZ on cell viability. MCF-7 and MDA-MB-231 cells were treated with 100 μM AT-I, 50 nM QNZ or their combination for 48 h, and MTT assay was adopted to determine cell viability. Significant differences between different groups were indicated as *p < 0.05, **p < 0.01, vs. control group, n = 3.

AT-I is a novel TLR4-antagonizing agent (Liu et al., 2016), and it exerted anti-tumor effects on colorectal cancer, bladder cancer and melanoma (Fu et al., 2018; Guo et al., 2019; Li et al., 2020), then we investigated whether it could suppress tumorigenesis in breast cancer via inhibiting TLR4/NF-κB pathway. Firstly, we found that AT-I could inhibit cell growth, proliferation, migration and invasion, and induce apoptosis in breast cancer cells. Then, we detected the effects of AT-I on the TLR4/NF-κB pathway in breast cancer cells. Previous studies have demonstrated that activation of TLR4 could lead to its dimerization, activation of the MyD88-dependent or -independent NF-κB signaling pathway, thus promoting tumor growth and invasion by regulating tumor immune and inflammatory response (Płóciennikowska et al., 2015; Chen et al., 2018). Furthermore, TLR4 could activate the downstream IκB kinase (IKK) complex, and then phosphorylate IκB, an NF-κB inhibitor that could prevent nuclear translocation of NF-κB. Upon phosphorylation, IκB was degraded and released NF-κB, which then entered nucleus and mediated the expression of inflammatory cytokines (Wang et al., 2015; Shi et al., 2019). In the present study, we found that the expression of TLR4, MyD88, p-NF-κB p65, p-IκBα and p-IKKα/β in breast cancer cells was significantly down-regulated after AT-I treatment. Furthermore, we used the LPS (TLR4 agonist) treatment as cell inflammation model, and measured the levels of secreted pro-inflammatory cytokines regulated by NF-κB. The results showed that the secretion of TNF-α, IL-6 and IL-1β were all decreased after AT-I treatment, and we concluded that AT-I could suppress tumorigenesis in breast cancer via inhibiting TLR4/NF-κB pathway, and down-regulate downstream pro-inflammatory cytokines. In addition, some studies suggested that TLR4 could activate both NF-κB and MAPK cascades via MyD88-dependent pathway (Ahmed et al., 2013). To verify the essential role of NF-κB in the inhibition of MyD88-dependent pathway by AT-I, we utilized an NF-κB inhibitor, QNZ to performed MTT assay in both MCF-7 and MDA-MB-231 cells. The cells were treated with 100 μM AT-I or 50 nM QNZ for 48 h, and the results (Figure 8) showed that AT-I and QNZ could inhibit MCF-7 and MDA-MB-231 cell proliferation, independently. However, compared with QNZ group, a combination of AT-I and QNZ couldn’t further inhibit cell proliferation, which indicated the important role of NF-κB in the effects of AT-I on breast cancer cell viability. Furthermore, it should be noted that only NF-κB inhibitor was used in our study, and other assays, such as using MAPK inhibitor or silencing both NF-κB and MAPK could better clarify the mechanism of AT-I effects.

TLR4, in the frame of our investigation, played a pivotal function role in the tumorigenesis inhibition of AT-I by regulating NF-κB signaling pathway. We found that LPS could induce cell migration and invasion, and co-treatment with AT-I or shTLR4 could decrease the migration and invasion. However, in TLR4 knockdown cells, no inhibitory effects of AT-I on cell migration and invasion were observed, which indicated that TLR4 was essential in AT-I’s effects. To further elucidate the underlying mechanisms, we examined the effects of TLR4 on the NF-κB signaling pathway and downstream pro-inflammatory cytokines, and we found that the expression of TLR4, MyD88, p-NF-κB p65, p-IκBα and p-IKKα/β and downstream pro-inflammatory cytokines TNF-α, IL-6 and IL-1β were significantly down-regulated after AT-I or shTLR4 treatment in LPS induced breast cancer cells. Similarly, in TLR4 knockdown cells, no inhibitory effects of AT-I on protein expression were observed, which indicated that AT-I’s effects on these protein levels were TLR4-dependent. The results above demonstrated that AT-I suppressed tumorigenesis in breast cancer cells via inhibiting TLR4-mediated NF-κB signaling pathway.

We further confirmed these effects and mechanisms *in vivo* using NMU-induced rat breast cancer model (Thompson et al., 1995), which shared a lot similarities with human mammary carcinomas including histopathology (Chan et al., 2005; Chan et al., 2007). Previously, we had used this model to explore the inhibitory effects of AT-II on breast cancer through regulating Nrf2/ARE pathway (Wang et al., 2017). In the present study, we found that AT-I could inhibit the mammary tumorigenesis in rats, and the underlying mechanisms were further elucidated by detecting the effects of AT-I on TLR4/NF-κB pathway, and its downstream proinflammatory factors in breast cancer rats. The results indicated that TLR4, MyD88 and p-NF-κB p65 were up-regulated in NMU-induced breast cancer, along with its downstream pro-inflammatory factors. While AT-I treatment could down-regulate TLR4/NF-κB pathway, and then decrease the TNF-α, IL-6 and IL-1β levels in NMU-induce rats. Our findings suggested that AT-I could inhibit NMU-induced mammary tumor progression in rats by inhibiting of TLR4/NF-κB pathway.

In summary, this study demonstrated that TLR4 and NF-κB were over expressed in breast cancer, and AT-I could suppress tumorigenesis of breast cancer via inhibiting TLR4-mediated NF-κB signaling pathway. These findings also provided proof that inhibiting TLR4/NF-κB pathway by natural compounds was an effective chemopreventive strategy for breast cancer, and AT-I appeared to have potential value as a novel candidate for breast cancer treatment.

## Data Availability Statement

The original contributions presented in the study are included in the article/Supplementary Material, further inquiries can be directed to the corresponding author.

## Ethics Statement

The animal study was reviewed and approved by Ethics Committee of Sichuan Provincial Maternity and Child Health Care Hospital.

## Author Contributions

TW designed the studies and wrote the manuscript. FL performed the experiments, analyzed data and wrote the manuscript. HL, XZ and JZ helped with the experiments. HX suggested experiments and revised the manuscript. All authors contributed to manuscript and reviewed and approved the manuscript.

## Funding

This work was mainly supported by the National Natural Science Foundation of China (81703922, 81803931), Basic Research Project of Health Commission of Sichuan Province (20PJ109), Basic Research Projects of Education Department of Sichuan Province (18ZB0164 and 18ZB0240) and Chengdu Key Research and Development Project (2019-YF05-01173-SN).

## Conflict of Interest

The authors declare that the research was conducted in the absence of any commercial or financial relationships that could be construed as a potential conflict of interest.
